# Letter to the Editor: Atypical duplex appendix arising from the ascending colon: a case report

**DOI:** 10.1186/s13256-024-04687-w

**Published:** 2024-07-31

**Authors:** B. M. Munasinghe

**Affiliations:** Ministry of Health, Colombo, Sri Lanka

**Dear Editor,**

We read with interest the article by Elsherbini *et al*. [1]. Previously, we reported probably the first case of appendix located in the ascending colon [2].

Developmental anomalies of the appendix are extremely rare [3]. However, they might lead to atypical presentations, atypical imaging findings, and missed or misdiagnosis. Sophisticated imaging modalities such as computed tomography have markedly improved preoperative diagnosis of such anomalies despite the relative scarcity in low-resource centers in developing countries such as ours. Detection of developmental anomalies in the appendix through clinical and ultrasonic evaluation is an interesting area to explore.

There was no duplex appendix in contrast to the report by Elsherbini *et al*. in our patient. There was no history suggestive of any previous episodes of unattended acute appendicitis with probable involution of a primary appendix in the cecum. This was further reinforced during the open laparotomy (as the laparoscope was out of order) with no local omental or bowel alterations associated with such an episode. Another possibility was the involution of the appendix with concurrent colonic diverticula. However, colonic diverticula are relatively uncommon, and most belong to the “false” category, devoid of the classical three layers of the intestinal wall.

We agree with Elsherbini *et al*. on the need for further expansion of the Cave–Wallbridge classification of the duplex appendix [4]. Given the increasing availability and utility of imaging and laparoscopic and endoscopic evaluation of abdominal pain, more atypical presentations of the appendix will probably be reported, thus the need for expanding the current classifications. In our case, the definite diagnosis of colonic appendix was only concluded with the histopathological analysis. The preoperative ultrasound study done by an experienced operator failed to identify the appendix in the usual position, and the abnormal position was concurrently not detected, amounting to a “false negative” scan. This is unsurprising given that ultrasonic detection of acute appendicitis is quoted around 76% [5].

We kindly would like to point out to the authors that the term “vermiform appendix” is used to identify the classical appendix originating from the confinements of the cecum. The duplex appendix thus should be referred to as “vermiform appendix duplex.” In addition, what we gathered from the case notes is that the first appendicectomy had been performed via an open approach (with a description of a right iliac fossa surgical scar). Once the classical anatomical and pathological features of appendicitis were detected, further exploration of the rest of the bowel might not have been carried out, thus missing the opportunity for concurrent detection of the duplex appendix. In our experience, it is common practice for surgeons to explore proximally to look for Meckel’s diverticulum during appendicular surgeries. Similarly, laparoscopy, compared with open surgery, offers more panoramic views and better visualization proximally and distally, increasing the likelihood of diagnosing other associated anomalies. The surgeon’s experience might also play a part in picking up such atypical appendicular pathologies, as appendicectomy is the most common surgical procedure performed by trainee surgeons.

Lastly, we would like to acknowledge the authors and congratulate them on successfully managing the patient and presenting their experience to their peers to widen the knowledge of a rare clinical entity.

## Data Availability

Data sharing does not apply to this article, as no datasets were generated or analyzed during the current study.
